# Geographic and Climatic Variation in Resin Components and Quality of *Pinus oocarpa* in Southern Mexico Provenances

**DOI:** 10.3390/plants13131755

**Published:** 2024-06-25

**Authors:** Mario Valerio Velasco-García, Adán Hernández-Hernández

**Affiliations:** 1 National Center for Disciplinary Research in Conservation and Improvement of Forest Ecosystems (Cenid Comef), National Institute of Forestry, Agriculture and Livestock Research (INIFAP), Ciudad de México 04010, Mexico; 2 Experimental Field Valles Centrales de Oaxaca, National Institute of Forestry, Agriculture and Livestock Research (INIFAP), Villa de Etla 68200, Mexico; hernandez.adan@inifap.gob.mx

**Keywords:** rosin, turpentine, acidity index, saponification index, total variance, correlation

## Abstract

In Mexico, there is a deficit in the production of pine resin, because it relies on natural forests only. Therefore, it is necessary to select provenances and phenotypes of potential species such as *P. oocarpa*. The objective was to determine the difference between provenances and the variation in resin components and quality, as well as the effect of geographic and climatic factors. Resin from five provenances was collected from southern Mexico. The percentage of rosin, turpentine and water was obtained, as well as the acidity and saponification index. *P. oocarpa* resin had 80.94% rosin, 7.7% turpentine and 11.49% water. The saponification and acidity index was 125.47 and 117.49 mg KOH.g^−1^, respectively. All variables showed differences (*p* ≤ 0.0001) between provenances. The provenance contributed between 6.44 and 11.71% to the total variance, the error contributed between 88.29 and 93.56%. Geographic and climatic variables only had an effect on the percentage of turpentine; the correlation was negative with altitude and longitude, but positive with temperature and precipitation. The results allow defining seed collection sites for resin plantations and orienting the selection for a *P. oocarpa* improvement program.

## 1. Introduction

In Mexico, pine resin is the most important non-timber plant product of forests, accounting for 39–44% of the annual national non-timber forest economic value [1]. Pine resin is commercially important because it is the source of important components such as rosin, the solid fraction, and turpentine, the volatile fraction [2,3]. Rosin is used mainly for adhesives, elastomers, printing inks, paper glues and paint, while turpentine is used mostly for solvents, pine oil, cleaners, aromas and vitamins [4,5]. More specifically, the resin is composed of resin acids, free fatty acids, combined fatty acids and unsaponifiables [6], which are mainly composed of terpenes, pinenes, limonenes and other chemical compounds useful for a variety of industries [3,6,7,8,9].

Pine resin production in Mexico depends mainly on natural forests, and the species of greatest production are *P. oocarpa* Schiede *ex* Schltdl., *P. devoniana* Lindl., *P. pringlei* Shaw, *P. montezumae* Lamb, *P. leiophylla* Schiede *ex* Schltdl. & Cham., *P. teocote* Schiede *ex* Schltdl. & Cham., *P. douglasiana* Martínez, *P. lawsonii* Roezl *ex* Gordon and *P. pseudostrobus* Lindl. [10,11]. Despite the variety of potential species for resin production in Mexico [12], demand is greater than production [10]; therefore, this raw material is imported from Venezuela, Honduras, China and Cuba [10]. An alternative to reverse the resin production deficit in Mexico is to develop forest genetic improvement (FGI) programs. Through FGI, it is possible to significantly increase resin production [13], due to the high genetic control of productivity [13,14] and other related characteristics [15]. Significant gains can be obtained from the beginning of the FGI process; for example, in *P. pinaster* Ait. during selection, phenotypes with a high resin yield (25.1 kg/year^−1^) compared to the average of control trees (7.3 kg/year^−1^) were found [14]. Genetic gains in resin production ranging from 8.0 to 17.5% are achieved by FGI [13,15].

Therefore, since 2010, public agencies and producers initiated research to improve resin production of *P*. *oocarpa*, *P. pringlei* and *P. pseudostrobus* in Michoacán, Mexico [15,16,17,18]. Among these species, *P. oocarpa* is the most important in resin production [1,12] and the most widely distributed conifer in Mexico [19]. *P. oocarpa* is characterized as a species with high resin productivity; for example, in Colombia it was demonstrated that its yield (80.79 kg ha^−2^ per month) exceeds that of *P. patula* Schiede *ex* Schltdl. (58.9 kg ha^−1^ per month) [20]. Resin production per individual tree of *P. oocarpa* in natural stands in southern Mexico is 2 to 3 kg, which is higher than that reported for other resinous species such as *P. sylvestris* L., *P. roxburghii* Sarg., *P. kesiya* Royle *ex* Gordon, *P. nigra* J. F. Arnold ssp. *laricio* Maire and *P. halepensis* Mill. [21]. Therefore, in 2019, the National Institute of Forestry, Agriculture, and Livestock Research initiated a broader research project to improve the resin production of this species in Mexico, through the selection of high-yield resin provenances and phenotypes [22,23].

In countries such as Mexico, where FGI for resin production is poorly developed [15], the first step is to select superior provenances and trees [24]. The study of provenances can provide insight into the levels and distribution of genetic variation, which is indispensable for genetic improvement [23]. The resin components (rosin and turpentine), quality and genetic gain of resin yield of *P. merkusii* Jungh. *et* de Vriese were different among provenances [13,25,26]. Likewise, the effect of provenance was observed in the amount of monoterpenes in *P. sylvestris* [27], and in the frequency and diameter of resin canals in *P. nigra* Arn. subsp. *salzmannii* (Dunal) Franco [28]. This highlights the importance of the precedence study for the improvement of resin species such as *P. oocarpa.*

In Mexico, although there are genetic improvement initiatives for resin production [15,16,17], there are no studies on the resin components (rosin and turpentine) and the quality of *P. oocarpa* resin. In other countries, it was determined that *P. oocarpa* resin contains between 75 and 82% rosin and 12.7 and 14.5% turpentine [5,29,30,31]. The resin quality of this species was also determined in other countries, the acidity index ranged from 133.0 to 143.9 mg KOH.g^−1^, whereas the saponification index ranged from 134.0 to 148.4 mg KOH.g^−1^ [5,29,30,31]. Knowledge of the resin components (rosin or turpentine) and the quality of the resin is important to guide its commercialization to suitable industries [2,5].

On the other hand, geographical and climatic variables of provenances influence resin productivity, resin chemical constitution, turpentine content and yield, and essential oil profile of some species such as *P. halepensis* and *P. merkusii*, *P. sylvestris*, *P. nigra* and *P. peuce* Griseb. [25,32,33,34,35,36]. For Mexican pine species and in particular for *P. oocarpa*, the relationship between resin characteristics with geographic and climatic variables has not been studied. However, geographic and environmental factors influence the number of cotyledons, seed emergence parameters and tree growth of this species [23,37,38]. Knowledge of the relationship between resin characteristics and geographic and climatic factors is important to guide the collection of seeds to establish resin-producing plantations [18,23].

Based on the above, the objective of this study was: (1) to determine the differences between provenances and the distribution of variation in the resin components and quality indicators of *P. oocarpa* resin in southern Mexico; (2) to determine the levels of association between geographic and climatic variables vs. resin components and quality indicators of resin. The research hypotheses were: (1) there are differences between provenances and the contribution of provenance to total variance will be high, due to the high geographic differentiation in these resin characteristics; (2) the association between geographic and climatic variables vs. resin variables will be significant because the latter are adaptive characteristics influenced by the environment of the site of origin.

## 2. Results

### 2.1. Differences between Provenances and Contribution to Variance 

Significant differences (*p* ≤ 0.0001) between provenances were found for all resin components and quality indices evaluated. In general, the resin of the *P. oocarpa* provenances evaluated contained an average of 80.94% rosin, 7.7% turpentine and 11.49% water. Among the provenances, El Tizne had the highest rosin content (83.71%), Sesteadero the highest turpentine content (9.65%) and San Pedro the lowest water content (9.7%), while El Nanche stood out among the provenances for having the lowest percentages of rosin and turpentine, and high water content (Table 1). The rest of the provenances presented intermediate values of rosin, turpentine and water content, but statistically different from the extreme values (Table 1). 

The average saponification index was 125.47 mg KOH.g^−1^ and the acidity index was 117.49 mg KOH.g^−1^. The highest and lowest values of the saponification index were found in San Pedro and Sesteadero, respectively. Likewise, Sesteadero presented the lowest value of the acidity index and the highest value was presented by the provenance Las Tejas (Table 1).

For both resin components and quality indices, the contribution of the provenance to the total variance was moderate, whereas the greatest proportion of the total variance was concentrated in the error (Table 2). As for the resin components, the average contribution of the provenances to the total variance was 9.02% and the error contributed 90.98%. The highest contribution of the provenances to the total variance occurred in the percentage of rosin (9.56%); while, the lowest contribution occurred in the percentage of turpentine (8.68%). On the other hand, the highest and lowest contribution of the error to the total variance occurred in the percentage of turpentine and percentage of water, respectively (Table 2). Regarding the resin quality indexes obtained (saponification index and acidity index), the provenances contributed an average of 9.07% to the total variance, and the error contributed an average of 90.93%. The highest and lowest contribution of the provenances to the total variance occurred in the saponification index (11.71%) and in the acidity index (6.44%), respectively; on the other hand, the contribution of the error was exactly the opposite for these indices (Table 2).

### 2.2. Provenance Clustering

**Figure 1:**
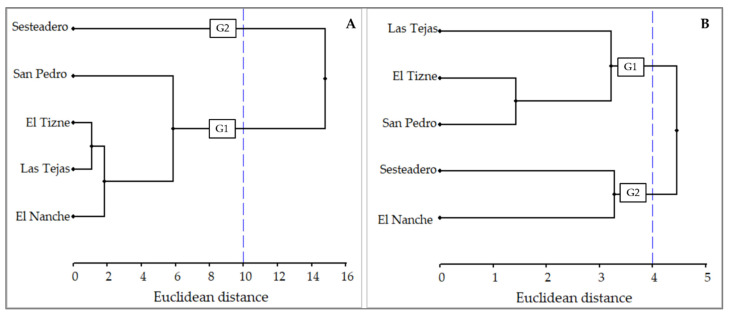
Grouping (G1: group one; G2: group two) of *Pinus oocarpa* provenances by geographic and environmental factors (**A**) and by resin components and quality (**B**).

The cluster analysis based on geographic and climatic factors revealed the formation of two groups; four provenances formed the first group (G1), while only the Sesteadero provenance formed the second group (G2) (Figure 1A). Similarly, the clustering based on resin components and quality indicators showed two groups, with Las Tejas, El Tizne, and San Pedro forming the first group, while Sesteadero and El Nanche formed the second group (Figure 1B).

### 2.3. Association between Geographic and Environmental Variables with Resin Components and Quality

The percentage of turpentine content of the resins was associated with most of the geographical and environmental variables of the origins. On the other hand, the percentage of rosin, percentage of water and the quality indexes (saponification index and acidity index) of resin were not associated with all the geographical and climatic variables (Table S1). 

**Figure 2:**
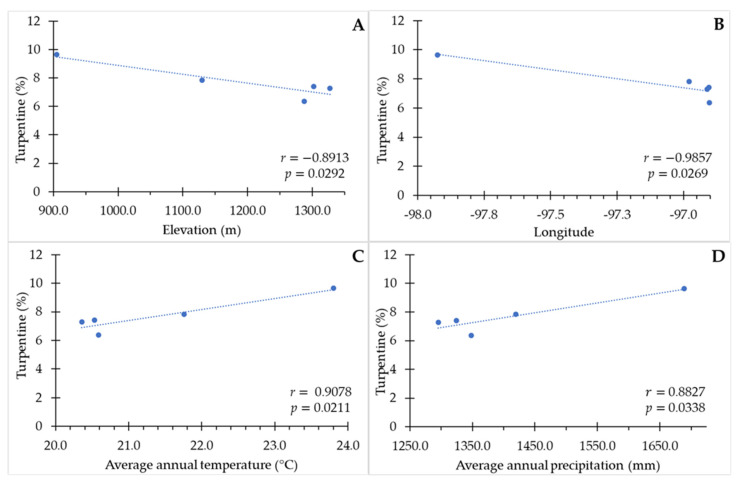
Relationship between elevation (**A**), longitude (**B**), mean annual temperature (**C**), and mean annual precipitation (**D**) with the percentage of turpentine.

The percentage of turpentine content of the resin had a high and significant negative association with the elevation (r = −0.8913, *p* = 0.0292) and longitude (r = −0.8957, *p* = 0.0269) of the provenances (Figure 2A,B). The relationship between turpentine content and latitude of the provenances was positive and high (r = 0.8532), but not significant (*p* = 0.0514). 

Likewise, turpentine content presented a significant association (*p* ≤ 0.0496) with all climatic variables; except with seven variables (sday: Julian date of the last freezing date of spring, ffp: Length of the frost-free period, winp: winter precipitation (nov + dec + jan + feb), adi: Annual dryness index (dd5/map), sdi: summer dryness index (dd5/gsp), ami: annual moisture index (qrt(dd5)/map), and smi: summer moisture index (gsdd5/gsp)) (Table S1). 

Turpentine content presented a high positive association with temperature (r = 0.9078, *p* = 0.0211) and precipitation (r = 0.8827, *p* = 0.0338) annual mean of the provenances (Figure 2C,D). Turpentine content was also positively correlated (r = 0.8743 to 0.9205; *p* = 0.0157 to 0.0385) with 12 other climatic variables, whereas, with three climatic variables, the correlation was negative (r = −0.8560 to −0.9113; *p* = 0.0195 to 0.0496) (Table S1). 

## 3. Discussion

### 3.1. Differences between Provenances and Contribution to Variance

The determination of resin components and quality is important for commercialization with industries that use this raw material for specific products [2]. The hypothesis of differences between provenances in the resin characteristics evaluated was accepted; this highlights the importance of the study of provenances to define seed sources [24,39] for resin-producing plantations. In this regard, if the aim is to produce resin with a high rosin content in México, seeds should be collected from the El Tizne provenance; conversely, if resin with a high turpentine content is desired, seeds from the Sesteadero provenance should be chosen. On the other hand, seeds from El Nanche are not recommended due to their high water content and low rosin and turpentine content. The San Pedro provenance is interesting because its resin has a lower water content and is in second place in percentage of rosin and turpentine content. Differences in resin components have been reported in Indonesian *P. merkusii* provenances [25] as well as between *P. halepensis* provenances in resin yield and essential oil composition [32]. Similarly, variations in the content of monoterpenes in *P. sylvestris* [27] resin, as well as in the frequency and diameter of resin canals in *P. nigra* subsp. *salzmannii* [28], are due to the effect of provenance.

The percentage of rosin of all the provenances studied was higher than that reported for plantations and natural forests of the same species (75%) in Colombia [5,31]. The percentage of rosin of the Sesteadero and El Nanche provenances was lower than that reported for the species in plantations (82.1%) in Brazil [29]; but the rest of the provenances studied presented higher values. On the contrary, the percentage of turpentine of all the provenances was lower than that reported (12.7 to 14.55%) for the same species [29,31]. Therefore, it can be affirmed that the rosin content of El Tizne, San Pedro, Las Tejas and Sesteadero is high in reference to previous studies [5,23,29]. 

Compared to other resinous pine species, the percentage of rosin of all *P. oocarpa* provenances was higher than that obtained for *P. caribaea* Morelet var. *caribaea* Bar. *et* Gol. (64 to 70%) in Cuba [2], *P. patula* (74.55%) in Colombia [4,31]. Likewise, the provenances of *P. merkusii* presented lower rosin content (72.0 to 78.0%) [25] and was only higher than the percentage determined for the El Nanche provenance of *P. oocarpa*. On the contrary, higher percentages of rosin were reported for *P. elliottii* Engelm var. *elliottii* (78.9%), *P. caribaea* Mor. var. *bahamensis* Bar. *et* Gol. (80.3%) and *P. kesiya* (87.3%) [29]. Meanwhile, the turpentine percentages of all provenances was lower than reported (14.55 to 20.00%) for other resin pine species [2,4,25,29,31]; except *P. kesiya*, which had lower percent turpentine (7.10%) [29] compared to most *P. oocarpa* provenances evaluated in this study.

Therefore, in general, it can be stated that the resin from the *P. oocarpa* provenances in southern Mexico are suitable for the production of rosin and would be used in the adhesives, elastomers, printing ink, chewing gum, soaps, detergents and paints industries [4,5,26]. 

Regarding resin quality indices, in general all provenances presented low values of saponification and acidity [4]. However, the differences between provenances in these parameters allow the selection of collection sites, depending on the type of industry that demands the resin. Sesteadero resin, being finer and less acidic, has a higher fraction of essential oils, so it can be used in the pharmaceutical industry [40]. This, in a way, is logical because the resin from this source had the highest proportion of turpentine. It is reported that turpentine from other populations of *P. oocarpa* in Mexico are suitable for the pharmaceutical industry because they have high contents of alpha pinene, beta pinene and L-limonene [41]. The resin from San Pedro and Las Tejas, being denser and more acidic, respectively, can be used for the manufacture of soaps, shampoos and conditioners [40]. Differences between provenances in acidity and saponification indices have also been found in *P. merkusii* [26].

The values of both indices, although lower, were close to the values reported for the same species in Colombia (SI = 134.0 to 137.35 mg KOH.g^−1^; AI = 133.89 to 135.0 mg KOH.g^−1^) [5,31]. The saponification index and acidity index values reported for *P. oocarpa* in Colombia indicated medium to high resin quality; therefore, according to the values obtained, the provenances analyzed in this study presented intermediate quality [31]. It has been indicated that the best quality resin, usually have acidity indices of 160 to 170 [4,31]; however, quality is relative and depends on the industry. Thus, resins with lower saponification and acidity values have a higher proportion of essential oils and are intended for the pharmaceutical industry; on the other hand, resins with high acidity and saponification values are important for cosmetic uses, production of shampoos, soaps and conditioners [40]. Therefore, resin industries have their own quality specifications depending on the product they produce [4]. The saponification and resin acidity index of the *P. oocarpa* provenances were similar to the values obtained in the resin (SI = 121.2 to 132.6 mg KOH.g^−1^; AI = 112.40 to 120.85 mg KOH.g^−1^) of *P. tropicalis* Morelet, *P. cubensis* Griseb., *P. occidentalis* Sw. and *P. caribaea* [42], which were found to be excellent for obtaining paper siccatives, industrial emulsifiers, disinfectants, mortar and concrete additives, and specialized lubricants [42]; therefore, *P. oocarpa* resin can also be used for these purposes.

Knowledge of the levels and structure of variation in traits of interest in forest trees is essential to guide selection efforts in forest higher values of both indices were obtained for *P. oocarpa* in plantations in Peru (AI = 198.0, SI = 291.91 mg KOH.g^−1^) [30]; however, these high values represent low resin quality if the purpose is to obtain resin oil [30]. Likewise, *P. caribaea* var. *caribaea*, *P. patula*, and *P. merkusii* present higher values of acidity and saponification (AI = 133.89 to 223.4 mg KOH.g^−1^, SI = 136.20 to 217.90 mg KOH.g^−1^) [2,4,31,43,44,45,46]. 

The low values of the acidity and saponification index of the *P. oocarpa* provenances, compared to the high values obtained in *P. oocarpa* plantations in Peru, are possibly because, in this research, no chemical stimulant was used during resin extraction. These indices were very high when chemical stimulant was used in the resin extraction process of other species [30,43,45]. For its part, the addition of maleic anhydrous acid increased the acidity and saponification values of *P. merkusii* resin [43]. Likewise, the oxidation of the resin increases the acidity and saponification values [4,30], due to the fact that the proportion of acids present in the resin is modified [31]. The resin collection method for this study was effective, as it did not allow contact with the external environment and contamination; therefore, it prevented oxidation. The differences between the results obtained and those of other species could be due to various factors, including interspecific effects, tree age and diameter, facing direction of the wounds, sample handling, resin collection system and timing, sampling duration, cultivation site, resin chemical compounds, and possibly genetic improvement of the species [4,5,30,41]. In countries with a tradition in resin production (e.g., China, Cuba and Brazil), higher yielding phenotypes have been selected [13,47,48,49]. Possibly the selection was oriented towards phenotypes with high acidity and saponification values with the aim of producing resin for the soap, shampoo and conditioner industry [40].

Knowledge of the levels and structure of variation in characteristics of interest in forest trees is essential to guide selection efforts in forest genetic improvement [24,39]. In general, the low contribution of provenance to the total variance of resin components (rosin, turpentine and water) and resin quality of *P. oocarpa* indicated a low degree of geographic differentiation for these traits. On the other hand, the high contribution of the error to the total variance indicated that the greatest variation was concentrated among trees within provenances, so that these characteristics (components and resin quality) have strong genetic control [50]. Genetic control was moderate to high for the density of resin canals in *P. oocarpa* trees of high resin productivity in populations of central Mexico [15]. Likewise, genetic control of resin production was high in *P. pinaster*, *P. massoniana* Lamb., *P. caribaea* Mor. var. *hondurensis* Bar. & Gol. and *P. merkusii* [13,14,25,48,49,51,52]. From the point of view of genetic improvement, the results of this research indicated that, in order to increase the genetic gain in resin components and quality, efforts should be oriented to select trees within each provenance [23,50].

The distribution of variation in resin characteristics in this study was similar to that of resin ducts in high-yielding *P. oocarpa* trees from other populations, where error had a high contribution (87.5 to 100%) to total variance [15]. In contrast, the low contribution of provenance to the total variance of resin characteristics was contrary to the high contribution of this factor (33.85 to 48.65%) to the total variance in seed emergence characteristics of the same trees [23]. This shows that sources of variation such as provenances and trees differentially influence resin and seed characteristics, possibly because there is no positive genetic correlation between seed and resin characteristics. However, for other species such as *P. massoniana*, *P. taeda* L. and *P. elliottii*, resin yield presented positive genetic correlation with normal diameter, height, volume, number of branches, number of whorls and crown variables [48,52,53]. This is important from the standpoint of forest genetic improvement; for *P. oocarpa*, selection should be directed at the provenance level or at the level of individual trees within provenances, depending on the trait to be improved.

### 3.2. Provenance Clustering

Clustering analyses allow us to graphically identify the similarities and dissimilarities of populations through their characteristics [54]. In this case, the analysis allowed us to know the similarity of the *P. oocarpa* provenances by geographical and climatic variables of the location, as well as by the resin components (rosin, turpentine and water) and quality of its resins. This can contribute to identify intraspecific geographic groups, varieties and define policies to preserve genetic diversity, as was the case of the study of the terpene composition of *P. nigra* [55].

The grouping by geographic and environmental factors indicated that the Sesteadero provenance is very different from the rest of the populations; possibly because it is located further west and at lower elevation, with higher temperature and mean annual precipitation. However, the grouping by resin components (rosin, turpentine and water) and resin quality did not correspond exactly with the grouping by geographic and climatic factors. In the grouping by resin characteristics, the Sesteadero and El Nanche populations, which are the most antagonistic populations in terms of elevation, temperature and precipitation, formed the same group. This indicates that the resin characteristics evaluated are not directly related to the joint variation in geographic and climatic factors. This may indicate that the variation in resin components and quality may be due to genetic factors [34] or phenotypic characteristics of the trees [20]. Even other variables such as soil type, geological substrate and exposure can influence resin characteristics such as yield and chemical components [16,34]. 

Contrary to this study, clustering by *P. oocarpa* seedling emergence parameters corresponded to clustering by geographic and climatic factors [23]. In contrast, for *P. peuce*, the clustering based on chemical components of resin essential oils did not correspond to the clustering based on bioclimatic factors [34].

### 3.3. Association between Geographic and Environmental Variables with Resin Components and Quality

The significant association between plant characteristics with elevation may represent adaptive response [38,56]. The correlation between percent turpentine with geographic and climatic factors is attributed to local adaptations [32]. Therefore, among the characteristics evaluated in this research, the percentage of turpentine content of *P. oocarpa* populations in southern Mexico may be an adaptive response because there is a pattern of clinal genetic differentiation; this pattern may be a consequence of a long-term process of natural selection, and therefore has evolutionary significance [35]. In contrast, the percentage of rosin content, as well as the acidity index and the saponification index possibly do not represent an adaptive response [38,56]. Similar to the results of this study, oleoresin yield of *P. halepensis* and *P. merkusii* decrease as elevation increases [25,32]; conversely, turpentine yield of *P. merkusii* is positively correlated with elevation [25].

The effect of elevation has been determined for other *P. oocarpa* traits, although not with the same pattern. Contrary to the negative relationship between elevation and percent turpentine, elevation was positively associated with germination parameters in the same provenances [23], which suggests that elevation has a differential influence on the traits in the same provenances. Likewise, in populations from Michoacán, Mexico, elevation was negatively associated with the number of cotyledons [37]. 

Longitude also had an effect on turpentine content, indicating that as the provenances are located further eastward, the turpentine content is lower. The effect of this geographical factor on resin characteristics and components of the *Pinus* genus has not been reported. On the contrary, the effect of latitude on the chemical constitution of *P. sylvestris* resin has been observed in Finland and Sweden [7,33,35]. In the foliage of *P. sylvestris* in Sweden, the number of tree individuals with higher amounts of limonene, B-pinene and 3-carene gradually increases with latitude [35]. In contrast, in Finland and Estonia, among nursery frown seedling of *P. sylvestris*, individual northern trees showed higher concentrations of total phenolics and monoterpenes, whereas individual resin acids, palustric and neoabietic acids, as well as some individual monoterpenes (limonene, tricyclene, camphene, f-pinene + sabinene and bornylacetate), were more common in northern seedlings [33]. In this investigation, although the correlation between latitude with turpentine percentage was high, it was not significant (r = 0.832, *p* = 0.0514). In Finland, in mature *P. sylvestris* trees, higher concentrations of pinosylvin, pinosylvin monomethylether and vanillic acid were found in the south, and, for example, lignans and pinosylvin monomethylether glycoside in the north [7]. In this study, regional differences were due to tree age and heartwood content, not directly the climatic factors (effective thermal sum) [7].

The significant relationship between the percentage of turpentine of *P. oocarpa* with climatic variables is logical due to the relationship between elevation and climate [23,38]; however, contrary to the negative relationship with elevation and latitude, most of the climatic variables with a significant relationship were positively related to the percentage of turpentine. This pattern of association between turpentine percentage with elevation (negative) and climatic variables (mostly positive) was contrary to the pattern found for seed emergence characteristics of the same provenances with elevation (positive) and environmental variables (mostly positive) [23]. The positive relationship between mean annual temperature and precipitation with the percentage of turpentine somehow coincided with what was reported in populations of *P. oocarpa* in Michoacán, Mexico, where it was determined that the semi-warm sub-humid climate and higher temperature favor resin production [16]. 

Similar to what was obtained in this study, the variability in *P. halepensis* resin chemistry depended on bioclimatic indices of temperature such as continentality index, ombrothermal index, summer precipitation for the three warmest consecutive summer months, and summer drought index [32]. Contrary to the relationship found in this research, the diameter of resin canals of *P. nigra* subsp. *salzmannii* was negatively correlated with temperature-related factors (average annual temperature, lowest average monthly temperature, highest average monthly temperature, average minimum temperature in the month with the lowest average) [28]; the frequency of resin canals was also negatively correlated with the absolute minimum temperature [28]. In *P. nigra*, resin yield was positively correlated with precipitation, but negatively correlated with mean, minimum and maximum air temperature; in contrast, resin yield of *P. sylvestris* was not correlated with these factors [36]. In addition, the essential oil profile of *P. peuce* resin was positively correlated with bioclimatic parameters, which was not the case for *P. heldreichii* Christ [34]. On the other hand, the diversity of resin acids in *P. pinaster* was also related to mean annual temperature, while the temporal variation in these acids was related to maximum wind intensity [57]. The identification of clinal variation in turpentine content due to geographic and climatic factors in *Pinus oocarpa* populations may be useful in conservation and genetic improvement programs [35].

In this study, rosin percentage, water percentage, and acidity and saponification indices did not present significant correlation with geographic and climatic variables possibly because these resin characteristics may have greater genetic control independent of climatic fluctuations [34,52]. On the other hand, it is possible that these characteristics of *P. oocarpa* resin are related to the geological substrate, type and texture of the soil, such as the composition of essential acids in *P. heldreichii* [34] and the diversity of resin acids in *P. pinaster* [57]. In Michoacán, Mexico, it was determined that chromic luvisol soil type is related to higher resin production of *P. oocarpa* [16]. Another explanation is that the relationship between percent rosin, percent water and resin quality indicators do not have a simple linear relationship with geographic and climatic factors [34]. *P. oocarpa* seedling growth presented a quadratic correlation with altitude and annual humidity index in populations of Michoacán, Mexico [38]. 

Likewise, the lack of relationship between rosin percentage and resin quality with geographic and climatic factors may be attributed to the existence of pleiotropy and genetic linkage disequilibrium [48,49,52], which may cause positive association between resin characteristics with growth variables [49]. These hypotheses should be tested in *P. oocarpa* in future research. In plantations of *P. oocarpa* in Peru, the saponification index presented significant linear correlation with tree diameter, but not for the acidity index [30]. For other species such as *P. caribaea* var. *hondurensis*, *P. massoniana* and *P. nigra*, resin yield was correlated with normal diameter, height, stem volume and number of live branches [36,48,49]. 

## 4. Materials and Methods

### 4.1. Provenances and Tree Selection 

**Figure 3:**
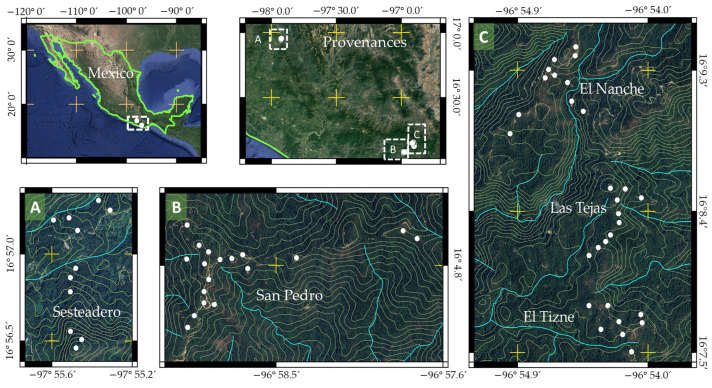
Location of *Pinus oocarpa* provenances in southern Mexico (Dotted line rectangles on the provenances map indicate the approximate location of the larger scale A, B, C maps; white dots indicate tree locations).

In 2019, five provenances of *P. oocarpa* were located in the southern Sierra of Oaxaca, Mexico (Figure 3; Table 3). These provenances were selected because they harbor healthy stands of the species, with potential for selecting trees with a high resin yield. The selection of provenances covered the altitudinal distribution range of the species in this region, which ranges from 1800 to 2400 m (Table 3). In each provenance, between 8 and 18 superior trees were selected, whose only indicator of selection was high resin production compared to trees in the same stand. 

Tree selection was carried out using the regression method, which consisted of two phases. The first phase consisted of the pre-selection of candidate trees and the second phase defined the superior trees. The pre-selection of candidate trees consisted of: (1) In healthy stands, phenotypes with wide crown, abundant foliage density, thick branches, larger normal stem diameter, greater volume and vigorous were visually located. These characteristics are related to greater resin production [58,59,60]; (2) Age, normal diameter, total height, canopy diameter and crown height were obtained for each tree. (3) The geographic location of each tree was recorded (altitude, latitude, longitude); these trees were called candidate trees. A total of 95 candidate trees were obtained; these were located more than 100 m linearly between them, to reduce the probability of kinship. The definition of superior trees consisted of: (1) The estimated resin production of each candidate tree was obtained with allometric equations [58]. (2) A scatter plot was obtained with the Excel^®^ software version 21H2; estimated resin production was the dependent variable and age the independent variable. (3) A simple linear regression line of the scatter points was obtained on the scatter plot. Trees located above the regression line were selected as superior trees. Out of 95 candidate trees, 67 top trees were obtained. Table 4 shows the dendrometric characteristics of the selected top trees.

### 4.2. Resin Collection and Analysis

**Figure 4:**
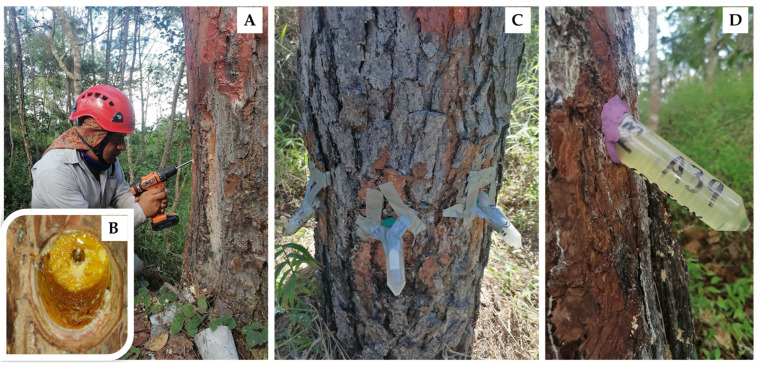
Illustration of *Pinus oocarpa* resin extraction method ((**A**): opening orifice, (**B**): detail of orifice, (**C**): installation of centrifuge tubes, and (**D**): centrifuge tube with resin before collection).

The resin was collected in November 2021, which is the beginning of the dry season of the year and when there is the greatest flow of resin [61]. In the stem of each tree, at a height of 80 cm from the ground, three to four holes were made to extract the resin. The holes were drilled with a 0.5-inch diameter drill and drill bit; this hole was 3 cm deep and inclined at approximately 30° (Figure 4A). Centrifuge tubes with a capacity of 50 mL were installed in each hole. The nozzle of the tubes was sealed with modeling clay to prevent resin loss, and the tubes were secured to the tree trunk with adhesive tape (Figure 4B,C). Resin samples were collected 48 h after installing the tubes. Between 100 and 150 mL of resin was collected from each tree. No stimulants were applied to the holes to accelerate queen extraction; therefore, in some trees additional tubes were installed to complete the required resin volume. The resin samples were transferred to the laboratory and stored in a refrigerator at 5 °C until processing.

The resin samples were subjected to a steam distillation process [5,62] to separate their components: rosin, turpentine and water. After separating each component, they were weighed using a digital scale with a precision of 0.01 g; subsequently, the percentage of each component was obtained. The quality of the resins was determined using the saponification and acidity indices. The saponification index was determined according to the international standard ASTM-D1980-87 [63], and the acidity index was obtained according to the international standard ASTM-D464-15 [64]. The equations to determine the saponification index (SI) and the acidity index (AI) were as follows [63,64].

SI = [(B − A)N × 56.1]/C(1)

AI = (VN × 56.1]/S(2)

In Equation (1), B is the acid required (0.5 M HCL) for titration of the blank (mL), A the acid required for titration of the sample (mL), N the normal of the acid and C the weight of the sample (g). In Equation (2), V the KOH solution required for titration (mL), N the normal of the KOH solution, and S the weight of the sample (g).

### 4.3. Geographic and Climatic Variables

Geographic location data (latitude, longitude and altitude) for each tree were obtained for all provenances. With the geographic data, climatic variables of ecological importance in plant distribution [65] were obtained from Research on Forest Climate Change website of Virginia Tech [66]. With some climatic data, aridity and annual and summer humidity indices were calculated [38,67]. These data are published in Table S1 of a previously published study on seed emergence of these trees [23], in a supplement (https://www.mdpi.com/article/10.3390/seeds3010001/s1, accessed on 6 May 2024). 

### 4.4. Statistical Analysis

Five thousand *bootstrap* were obtained for each of the resin components variables (percentage of rosin, turpentine and water) and resin quality indexes (saponification index and acidity index) in each provenance (Table S2). The assumptions of normality and homogeneity of variances of data were verified with the Kolmogorov–Smirnov and Levene tests, respectively. No variable met both assumptions (*p* ≤ 0.01). Therefore, to know the differences between provenances, analysis of variance and RT-1 multiple comparisons were performed [68]. To determine the contribution of the provenances to the total variance in each variable, the variance components were estimated using the VARCOMP procedure and the RELM option of SAS [69], using the following statistical model:

(3) $$Y_{ij}=\mu +P_{i}+\varepsilon_{ij}$$

where *Y_ij_* is the observation value, *μ* is the population mean, *P_i_* is the effect of provenance, and *ε*_*i*_*_j_* is the experimental error.

To determine the similarity between the provenances, a clustering analysis was carried out according to geographical and climatic variables. Another grouping was obtained according to the resin components (rosin, turpentine and water) and quality indexes (saponification and acidity index) of the resin. The idea was to observe the correspondence between these groupings. These analyses were performed with Ward’s hierarchical clustering and Euclidean distance [54].

To evaluate the association between resin components and quality indexes with geographic and climatic variables, Pearson correlation coefficients were obtained from the average values of the provenances, using Fisher’s Z-transform. The average of resin components and quality was obtained from 5000 bootstrap, while the average of geographical and climatic variables was obtained from the original values (8 to 18 trees per provenance). All analyses were conducted using the statistical software SAS 9.4 [69].

## 5. Conclusions

The provenance factor influences the percentages of resin components and quality indices of *P. oocarpa*. The greatest variability in these resin characteristics is found among trees within provenances, while a smaller proportion of the variability is found among provenances; therefore, selection efforts should be concentrated among trees within provenances. Variation in the percentage of turpentine is associated with geographical and climatic factors; this is not the case for other resin components and quality. The higher the altitude and longitude, the lower the percentage of turpentine; the higher the temperature and average annual rainfall, the higher the percentage of turpentine. Knowledge of the resin components and the quality of the resin allows defining its commercialization to specific industries. Knowledge of the effect of origin, levels of variation, and the effect of geographic and climatic factors allows defining germplasm collection sites for resin plantations and directing selection efforts to continue with the improvement of *P. oocarpa*.

## Figures and Tables

**Table 1:** Resin components and quality indices of *Pinus oocarpa* in five provenances in southern Mexico.

Provenance	*N* ^1^	Resin Components (%)						Saponification Index (mg KOH.g^−1^)		Acidity Index (mg KOH.g^−1^)	
		Rosin		Turpentine		Water					
El Nanche	5000	76.47	d ^2^	7.29	d	16.36	d	127.06	c	119.59	b
Las Tejas	5000	82.46	b	7.41	c	10.22	b	121.78	b	109.28	d
El Tizne	5000	83.71	a	6.37	e	10.00	c	127.79	c	118.03	c
San Pedro	5000	82.58	b	7.83	b	9.70	a	129.49	d	118.65	b
Sesteadero	5000	79.49	c	9.65	a	11.16	c	121.25	a	121.89	a
Average	5000	80.94		7.71		11.49		125.47		117.49	

^1^ Number of Bootstrap generated from between 8 and 18 original samples in each provenance. ^2^ Different letters in each column indicate differences between origins (α ≤ 0.05).

**Table 2:** Variance components of resin components and quality of *Pinus oocarpa*.

Variable	*p* Value	Contribution to Total Variance (%)		Total Variance
		Provenance	Error	
Rosin percentage	<0.0001	9.56	90.44	90.73
Turpentine percentage	<0.0001	8.68	91.32	16.76
Water percentage	<0.0001	8.82	91.18	87.22
Saponification index	<0.0001	11.71	88.29	118.17
Acidity index	<0.0001	6.44	93.56	359.15

**Table 3:** Environmental data and number of selected *Pinus oocarpa* superior trees in five provenances in southern Mexico (MAT: mean annual temperature; MAP: mean annual precipitation).

Provenance	Community	Elevation (m)	MAT (°C)	MAP (mm)	Number of Trees	Identification Key of Trees
Sesteadero	Putla Villa de Guerrero	905.4	23.80	1688.64	11	SES01, SES02, SES03, …, SES11
San Pedro	San Domingo Coatlán	1129.8	21.76	1419.83	18	SDC30, SDC31, SDC32, …, SDC47
El Tizne	San Domingo Coatlán	1287.6	20.59	1348.13	8	SDC11, SDC12, SDC13, …, SDC18
Las Tejas	San Domingo Coatlán	1301.8	20.53	1324.70	10	SDC01, SDC02, SDC03 …, SDC10
El Nanche	San Domingo Coatlán	1327.2	20.36	1295.44	10	SDC19, SDC21, SDC22, …, SDC29

**Table 4:** Average age and dendrometric characteristics of *Pinus oocarpa* trees in southern Mexico.

Provenance	Number of Trees	Age (Years)	Normal Diameter (cm)	Total Height (m)	Crown Diameter (m)	Crown Length (m)	Crown Volume (m^3^)
Sesteadero	11	71.6	53.21	25.73	10.44	16.15	1014.17
San Pedro	18	73.3	47.47	16.86	9.58	11.47	652.30
El Tizne	8	53.6	43.63	19.74	10.61	12.34	804.31
Las Tejas	10	59.0	46.16	22.80	10.27	14.51	883.06
El Nanche	10	60.9	44.74	18.10	10.05	11.00	623.25

## Data Availability

Data are contained within the Supplementary Materials and in https://www.mdpi.com/article/10.3390/seeds3010001/s1 (accessed on 6 May 2024).

## Supplementary Materials

The following supporting information can be downloaded at https://www.mdpi.com/article/10.3390/plants13131755/s1, Table S1: Pearson correlation statistics (Fisher Z transformation) between geographical and climatic variables versus resin variables (components and quality) of *Pinus oocarpa* provenances in southern Mexico; Table S2: Bootstrap data resin components (rosin, turpentine and water) and quality (saponification index and acidity index) of *Pinus oocarpa* provenances from southern Mexico.
