# Supplementary material for: Geographic and Climatic Variation in Resin Components and Quality of Pinus oocarpa in Southern Mexico Provenances
Source: Plants (Basel). 2024 Jun 25;13(13):1755. doi: 10.3390/plants13131755 (PMC11244295; doi:10.3390/plants13131755)
Supplement: Supplementary file 1 [file plants-13-01755-s001.zip › Table S1.pdf]

Table S1. Pearson correlation statistics (Fisher Z transformation) between geographical and climatic variables versus resin variables (components and quality) of *Pinus oocarpa* provenances in southern Mexico.

Geographic and climatic variables[1]	Resin variables	Fisher's Z	Estimated correlation	95 % Confidence limits		*p* value
**elev**	**Rosin (%)**	0.0676	0.0591	-0.8685	0.8947	0.9239
**long**	**Rosin (%)**	0.2696	0.2324	-0.8175	0.9250	0.7030
**lat**	**Rosin (%)**	-0.3255	-0.2786	-0.9318	0.8004	0.6453
**mat**	**Rosin (%)**	-0.1062	-0.0927	-0.9013	0.8599	0.8806
**map**	**Rosin (%)**	-0.0943	-0.0823	-0.8993	0.8626	0.8940
**gsp**	**Rosin (%)**	-0.1304	-0.1137	-0.9052	0.8543	0.8537
**mtcm**	**Rosin (%)**	-0.0607	-0.0531	-0.8935	0.8699	0.9316
**mmin**	**Rosin (%)**	-0.0278	-0.0243	-0.8875	0.8768	0.9687
**mtwm**	**Rosin (%)**	-0.1621	-0.1411	-0.9101	0.8466	0.8187
**mmax**	**Rosin (%)**	-0.2174	-0.1883	-0.9181	0.8322	0.7585
**sday**	**Rosin (%)**	0.0884	0.0772	-0.8639	0.8983	0.9005
**fday**	**Rosin (%)**	0.1116	0.0974	-0.8587	0.9021	0.8746
**ffp**	**Rosin (%)**	0.0311	0.0272	-0.8761	0.8881	0.9650
**dd5**	**Rosin (%)**	-0.1054	-0.0920	-0.9011	0.8601	0.8815
**gsdd5**	**Rosin (%)**	-0.1095	-0.0956	-0.9018	0.8591	0.8770
**d100**	**Rosin (%)**	0.0768	0.0671	-0.8665	0.8963	0.9135
**smrpb**	**Rosin (%)**	-0.1849	-0.1606	-0.9134	0.8408	0.7937
**smrsprpb**	**Rosin (%)**	-0.2328	-0.2014	-0.9202	0.8280	0.7420
**sprp**	**Rosin (%)**	0.3121	0.2676	-0.8046	0.9302	0.6590
**smrp**	**Rosin (%)**	-0.1694	-0.1473	-0.9112	0.8447	0.8107
**winp**	**Rosin (%)**	0.8249	0.6293	-0.5688	0.9719	0.2434
**adi**	**Rosin (%)**	-0.0478	-0.0418	-0.8912	0.8727	0.9461
**ami**	**Rosin (%)**	-0.0287	-0.0251	-0.8877	0.8766	0.9676
**sdi**	**Rosin (%)**	0.1245	0.1086	-0.8556	0.9042	0.8602
**smi**	**Rosin (%)**	0.1086	0.0948	-0.8594	0.9016	0.8780
**Elev**	**Turpentine (%)**	-1.5423	-0.8913	-0.9928	-0.0423	0.0292
**long**	**Turpentine (%)**	-1.5645	-0.8957	-0.9931	-0.0640	0.0269
**lat**	**Turpentine (%)**	1.3777	0.8532	-0.1177	0.9901	0.0514
**mat**	**Turpentine (%)**	1.6308	0.9078	0.1284	0.9940	0.0211
**map**	**Turpentine (%)**	1.5008	0.8827	0.0017	0.9922	0.0338
**gsp**	**Turpentine (%)**	1.5296	0.8887	0.0299	0.9927	0.0305
**mtcm**	**Turpentine (%)**	1.5219	0.8871	0.0224	0.9925	0.0314
**mmin**	**Turpentine (%)**	1.4631	0.8743	-0.0351	0.9916	0.0385
**mtwm**	**Turpentine (%)**	1.6966	0.9186	0.1914	0.9947	0.0164
**mmax**	**Turpentine (%)**	1.7090	0.9205	0.2032	0.9948	0.0157

**sday**	**Turpentine (%)**	0.8330	0.6338	-0.5636	0.9724	0.2388
**fday**	**Turpentine (%)**	-1.5275	-0.8883	-0.9926	-0.0279	0.0308
**ffp**	**Turpentine (%)**	-1.3122	-0.8349	-0.9888	0.1799	0.0635
**dd5**	**Turpentine (%)**	1.6217	0.9063	0.1196	0.9939	0.0218
**gsdd5**	**Turpentine (%)**	1.6244	0.9067	0.1222	0.9939	0.0216
**d100**	**Turpentine (%)**	-1.3885	-0.8560	-0.9903	0.1073	0.0496
**smrpb**	**Turpentine (%)**	1.6050	0.9033	0.1034	0.9937	0.0232
**smrsprpb**	**Turpentine (%)**	1.5673	0.8962	0.0667	0.9932	0.0267
**sprp**	**Turpentine (%)**	-1.6511	-0.9113	-0.9942	-0.1480	0.0195
**smrp**	**Turpentine (%)**	1.5542	0.8937	0.0539	0.9930	0.0280
**winp**	**Turpentine (%)**	0.2170	0.1880	-0.8323	0.9180	0.7589
**adi**	**Turpentine (%)**	-0.8177	-0.6252	-0.9716	0.5733	0.2475
**ami**	**Turpentine (%)**	-1.0408	-0.7369	-0.9812	0.4156	0.1410
**sdi**	**Turpentine (%)**	-0.9050	-0.6724	-0.9758	0.5159	0.2006
**smi**	**Turpentine (%)**	-1.0907	-0.7578	-0.9829	0.3755	0.1229
**elev**	**Water (%)**	0.3042	0.2611	-0.8071	0.9293	0.6671
**long**	**Water (%)**	0.0862	0.0753	-0.8644	0.8979	0.9030
**lat**	**Water (%)**	-0.0164	-0.0143	-0.8854	0.8791	0.9816
**mat**	**Water (%)**	-0.2656	-0.2291	-0.9245	0.8187	0.7072
**map**	**Water (%)**	-0.2687	-0.2317	-0.9249	0.8177	0.7039
**gsp**	**Water (%)**	-0.2303	-0.1993	-0.9198	0.8287	0.7446
**mtcm**	**Water (%)**	-0.3108	-0.2666	-0.9301	0.8050	0.6602
**mmin**	**Water (%)**	-0.3449	-0.2944	-0.9341	0.7941	0.6257
**mtwm**	**Water (%)**	-0.2070	-0.1795	-0.9166	0.8350	0.7697
**mmax**	**Water (%)**	-0.1486	-0.1294	-0.9080	0.8499	0.8336
**sday**	**Water (%)**	-0.3916	-0.3319	-0.9392	0.7783	0.5797
**fday**	**Water (%)**	0.2541	0.2194	-0.8220	0.9230	0.7194
**ffp**	**Water (%)**	0.3282	0.2808	-0.7995	0.9321	0.6425
**dd5**	**Water (%)**	-0.2660	-0.2293	-0.9245	0.8186	0.7068
**gsdd5**	**Water (%)**	-0.2613	-0.2254	-0.9239	0.8199	0.7117
**d100**	**Water (%)**	0.2781	0.2394	-0.8150	0.9261	0.6941
**smrpb**	**Water (%)**	-0.1765	-0.1534	-0.9122	0.8429	0.8029
**smrsprpb**	**Water (%)**	-0.1237	-0.1079	-0.9041	0.8558	0.8611
**sprp**	**Water (%)**	0.0500	0.0437	-0.8722	0.8916	0.9436
**smrp**	**Water (%)**	-0.1895	-0.1646	-0.9141	0.8396	0.7887
**winp**	**Water (%)**	-1.1211	-0.7699	-0.9839	0.3503	0.1129
**adi**	**Water (%)**	0.3269	0.2797	-0.8000	0.9320	0.6439
**ami**	**Water (%)**	0.3622	0.3084	-0.7884	0.9360	0.6085
**sdi**	**Water (%)**	0.1526	0.1329	-0.8489	0.9086	0.8292
**smi**	**Water (%)**	0.2036	0.1766	-0.8359	0.9161	0.7734
**elev**	**Saponification index**	0.3942	0.3340	-0.7773	0.9394	0.5772

**long**	**Saponification index**	0.6908	0.5483	-0.6469	0.9642	0.3286
**lat**	**Saponification index**	-0.8018	-0.6161	-0.9707	0.5831	0.2568
**mat**	**Saponification index**	-0.4438	-0.3728	-0.9444	0.7592	0.5303
**map**	**Saponification index**	-0.5257	-0.4345	-0.9519	0.7261	0.4572
**gsp**	**Saponification index**	-0.5678	-0.4649	-0.9553	0.7076	0.4220
**mtcm**	**Saponification index**	-0.3723	-0.3165	-0.9371	0.7849	0.5986
**mmin**	**Saponification index**	-0.3612	-0.3076	-0.9359	0.7887	0.6095
**mtwm**	**Saponification index**	-0.5266	-0.4352	-0.9520	0.7257	0.4564
**mmax**	**Saponification index**	-0.6061	-0.4918	-0.9583	0.6898	0.3914
**sday**	**Saponification index**	0.1022	0.0892	-0.8608	0.9006	0.8851
**fday**	**Saponification index**	0.2894	0.2489	-0.8116	0.9275	0.6823
**ffp**	**Saponification index**	0.1834	0.1593	-0.8412	0.9132	0.7954
**dd5**	**Saponification index**	-0.4451	-0.3739	-0.9446	0.7586	0.5290
**gsdd5**	**Saponification index**	-0.4681	-0.3915	-0.9468	0.7497	0.5079
**d100**	**Saponification index**	0.5746	0.4697	-0.7045	0.9559	0.4165
**smrpb**	**Saponification index**	-0.6090	-0.4938	-0.9585	0.6884	0.3891
**smrsprpb**	**Saponification index**	-0.6623	-0.5297	-0.9623	0.6619	0.3490
**sprp**	**Saponification index**	0.6887	0.5470	-0.6480	0.9640	0.3301
**smrp**	**Saponification index**	-0.6082	-0.4932	-0.9584	0.6888	0.3897
**winp**	**Saponification index**	0.2961	0.2545	-0.8096	0.9283	0.6754
**adi**	**Saponification index**	0.7187	0.5660	-0.6317	0.9659	0.3095
**ami**	**Saponification index**	0.0248	0.0217	-0.8774	0.8870	0.9720
**sdi**	**Saponification index**	0.8278	0.6309	-0.5669	0.9721	0.2417
**smi**	**Saponification index**	0.7344	0.5758	-0.6229	0.9669	0.2990
**elev**	**Acidity index**	-0.6284	-0.5071	-0.9599	0.6789	0.3742
**long**	**Acidity index**	-0.5931	-0.4827	-0.9573	0.6959	0.4016
**lat**	**Acidity index**	0.5425	0.4468	-0.7188	0.9533	0.4430
**mat**	**Acidity index**	0.6271	0.5062	-0.6796	0.9598	0.3751
**map**	**Acidity index**	0.6066	0.4921	-0.6895	0.9583	0.3910
**gsp**	**Acidity index**	0.6035	0.4900	-0.6910	0.9581	0.3934
**mtcm**	**Acidity index**	0.6321	0.5096	-0.6771	0.9602	0.3713
**mmin**	**Acidity index**	0.6154	0.4982	-0.6853	0.9590	0.3841
**mtwm**	**Acidity index**	0.6234	0.5037	-0.6814	0.9595	0.3780
**mmax**	**Acidity index**	0.6090	0.4938	-0.6884	0.9585	0.3891
**sday**	**Acidity index**	0.5256	0.4344	-0.7262	0.9519	0.4573
**fday**	**Acidity index**	-0.6894	-0.5474	-0.9641	0.6476	0.3296
**ffp**	**Acidity index**	-0.6355	-0.5119	-0.9604	0.6754	0.3688
**dd5**	**Acidity index**	0.6289	0.5074	-0.6787	0.9599	0.3738
**gsdd5**	**Acidity index**	0.6225	0.5030	-0.6818	0.9595	0.3787
**d100**	**Acidity index**	-0.5795	-0.4732	-0.9562	0.7022	0.4125
**smrpb**	**Acidity index**	0.6017	0.4888	-0.6918	0.9579	0.3948

**smrsprpb**	**Acidity index**	0.5965	0.4851	-0.6943	0.9576	0.3989
**sprp**	**Acidity index**	-0.6024	-0.4893	-0.9580	0.6915	0.3942
**smrp**	**Acidity index**	0.5997	0.4874	-0.6928	0.9578	0.3964
**winp**	**Acidity index**	0.2206	0.1911	-0.8313	0.9185	0.7550
**adi**	**Acidity index**	-0.4051	-0.3426	-0.9406	0.7735	0.5668
**ami**	**Acidity index**	-0.5866	-0.4782	-0.9568	0.6990	0.4068
**sdi**	**Acidity index**	-0.4333	-0.3647	-0.9434	0.7631	0.5401
**smi**	**Acidity index**	-0.5074	-0.4210	-0.9503	0.7338	0.4730

[1] mat: Mean annual temperature degrees C, map: Mean annual precipitation, gsp: Growing season precipitation, April to September, mtcm: Mean temperature in the coldest month degrees C, mmin: Mean minimum temperature in the coldest month degrees C, mtwm: Mean temperature in the warmest month degrees C, mmax: Mean maximum temperature in the warmest month degrees C, sday: Julian date of the last freezing date of spring, fday: Julian date of the first freezing date of autumn, ffp: Length of the frost-free period, dd5: Degree-days >5 degrees C (based on mean monthly temperature), gsdd5: Degree-days >5 degrees C accumulating within the frost-free period, d100: Julian date the sum of degree-days >5 degrees C reaches 100, dd0: Degree-days <0 degrees C (based on mean monthly temperature), mmindd0: Degree-days <0 degrees C (based on mean minimum monthly temperature), smrpb: Summer precipitation balance: (jul+aug+sep)/(apr+may+jun), smrsprpb: Summer/Spring precipitation balance: (jul+aug)/(apr+may), sprp: Spring precipitation (apr+may), smrp: Summer precipitation (jul+aug), winp: Winter precipitation (nov+dec+jan+feb), adi: Annual dryness index (dd5/map), ami: annual moisture index (qrt(dd5)/map), sdi: Summer dryness index (dd5/gsp), smi: summer moisture index (gsdd5/gsp).
